# Sinensetin: An Insight on Its Pharmacological Activities, Mechanisms of Action and Toxicity

**DOI:** 10.3389/fphar.2020.553404

**Published:** 2021-01-20

**Authors:** Lee Han Jie, Ibrahim Jantan, Syaratul Dalina Yusoff, Juriyati Jalil, Khairana Husain

**Affiliations:** ^1^ Drug and Herbal Research Centre, Faculty of Pharmacy, Universiti Kebangsaan Malaysia, Kuala Lumpur, Malaysia; ^2^ Institute of Systems Biology, Universiti Kebangsaan Malaysia, Bangi, Malaysia

**Keywords:** sinensetin, pharmacological activities, mechanistic studies, anticancer, anti-inflammatory, lipolysis

## Abstract

Sinensetin, a plant-derived polymethoxylated flavonoid found in *Orthosiphon aristatus* var. *aristatus* and several citrus fruits, has been found to possess strong anticancer activities and a variety of other pharmacological benefits and promising potency in intended activities with minimal toxicity. This review aims to compile an up-to-date reports of published scientific information on sinensetin pharmacological activities, mechanisms of action and toxicity. The present findings about the compound are critically analyzed and its prospect as a lead molecule for drug discovery is highlighted. The databases employed for data collection are mainly through Google Scholar, PubMed, Scopus and Science Direct. *In-vitro* and *in-vivo* studies showed that sinensetin possessed strong anticancer activities and a wide range of pharmacological activities such as anti-inflammatory, antioxidant, antimicrobial, anti-obesity, anti-dementia and vasorelaxant activities. The studies provided some insights on its several mechanisms of action in cancer and other disease states. However, more detail mechanistic studies are needed to understand its pharmacological effects. More *in vivo* studies in various animal models including toxicity, pharmacokinetic, pharmacodynamic and bioavailability studies are required to assess its efficacy and safety before submission to clinical studies. In this review, an insight on sinensetin pharmacological activities and mechanisms of action serves as a useful resource for a more thorough and comprehensive understanding of sinensetin as a potential lead candidate for drug discovery.

## Introduction

**FIGURE 1:**
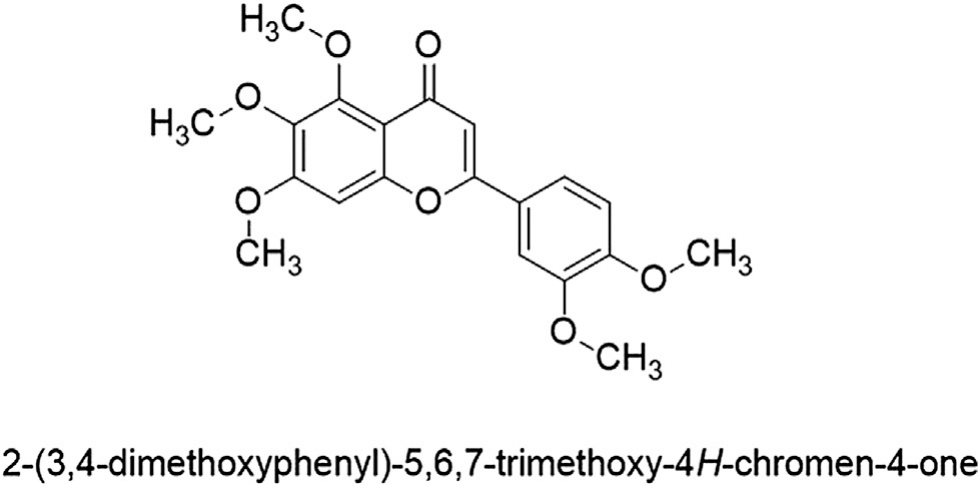
Structure of sinensetin.

Sinensetin is a polymethoxylated flavonoid found in *Orthosiphon aristatus* var. *aristatus* [syn: *Orthosiphon stamineus* Benth., *Orthosiphon spicatus* (Thunb.) Backer, Bakh.f. & Steenis] (Lambiaceae) and in many citrus fruits. In citrus fruits, it is particularly more abundant in the peel than other parts of the fruit (Manthey and Grohmann, 2001; Youn et al., 2017). A polymethoxylated flavonoid has a double bond between positions 2 and 3 and a ketone in position 4 of the C ring (Panche et al., 2016). Sinensetin is a pentamethoxyflavone, chemically known as 2-(3,4-dimethoxyphenyl)-5,6,7-trimethoxy-4*H*-1-benzopyran-4-one. The flavone skeleton is substituted by methoxy groups at positions 5, 6, 7, 3′ and 4’, respectively, with a molecular formula of C_20_H_20_O_7_ and molecular weight of 372.4 g/mol. Figure 1 shows the chemical structure of sinensetin.

*Orthosiphon aristatus* is widely employed in traditional medicine as a diuretic (Beaux et al., 1999; Adam et al., 2009) and to treat hypertension (Mohamed et al., 2012; Manshor et al., 2013), rheumatism, tonsillitis, gout, menstrual disorder and diabetes (Adam et al., 2009; Mohamed et al., 2012). The leaves of *O. aristatus* have been shown to exhibit excellent pharmacological activities such as antioxidant (Awale et al., 2003), antibacterial (Ho et al., 2014), hepatoprotective (Yam et al., 2007), anti-inflammatory (Hsu et al., 2010), cytotoxic (Yam et al., 2007), antihypertensive (Azizan et al., 2012) and gastroprotective (Yam et al., 2009). Many citrus fruits have been used traditionally for many medicinal purposes. For instance, fruit peels of *Citrus reticulata* Blanco [syn: *Citrus sunki* Hort. ex Tanaka, *Citrus unshiu* (Yu.Tanaka ex Swingle), *Citrus depressa* Hayata, *Citrus deliciosa* Ten.] have been used in Asian ethnomedicine to treat indigestion, bronchial, fever, snakebite, stomachache, edema, cardiac diseases, bronchitis and asthmatic conditions (Kang et al., 2013). Moreover, many *Citrus* species among the different variety of *C. reticulate* are recognized as food supplements and nutrition while their leaf extracts and essential oils have shown strong pharmacological activities such as antimicrobial (Tao et al., 2014), anti-inflammatory (Nasri et al., 2017), antioxidant (Nasri et al., 2017), anticancer (Benavente-Garcia O and Castillo, 2008), antiproliferative (Du, and Chen, 2010), hypoglycemic (Aruoma et al., 2012) and analgesic effects (Nasri et al., 2017).

There were several reports on the isolation of sinensetin from *O. aristatus.* Tezuka et al. (2000) reported the isolation of 75 mg of sinensetin from 4 kg of plant material by subjecting its methanol extract to repeated column chromatography followed by preparative thin layer chromatography (TLC). However, lower yields were obtained by other workers. Yuliana et al. (2009) reported 3.03 mg of sinensetin was isolated from 500 g of *O. aristatus* using silica column, sephadex LH-20 column and preparative TLC. Another study reported that from 1 kg of *O. aristatus*, only 2.6 mg of the compound was isolated by repeated column chromatography followed by preparative TLC (Amzad Hossain and Mizanur Rahman, 2015). The variable yields of sinensetin isolated by these workers might probably be due to the different separation techniques employed and other factors such as climate variability, soil conditions and geographical factors. Various yields of sinensetin were obtained from different *Citrus* species. Out of 100 mg ethyl acetate extract of *C. reticulata*, 23.4 mg sinensetin was isolated by using sephadex LH-20 column chromatography and preparative TLC (Nakanishi et al., 2019). The peels of *C. reticulata* (500 g of dry weight) yielded 38.8 mg of sinensetin by using silica gel column chromatography (Nagase et al., 2005). *Citrus reticulata* subjected to high-speed counter current chromatography (HSCCC) separation and preparative high performance liquid chromatography (HLPC) yielded 27.7 mg of sinensetin (Du and Chen, 2010). Polymethoxyflavone-enriched extract of Sweet Portuguese oranges (Newhall variety), obtained by supercritical fluid technology contained sinensetin at 17.36 mg/g as determined by HPLC with diode array detection (HPLC-UV/DAD) (Pereira et al., 2019). Sinensetin was the second most abundant methoxylated flavonoids isolated from 1 kg peels of *C. sinensis* (L.) Osbeck (Malterud and Rydland, 2000). Iwase et al. (2000), reported that from dried peels (955 g) of *C. sinensis* 13.4 mg sinensetin was isolated. Sinensetin is also available as a synthetic compound as it can be commercially prepared. Amzad Hossain and Ismail (2004) reported synthesis of sinensetin in high yield by alkaline condensation of 2,4,5,6-tetrahydroxyacetophenone and 3, 4-di (methoxymethoxy) benzaldehyde to give 2′, 4′, 5′, 6′, 3, 4-hexa (methoxymethoxy) chalcone, followed by dimethoxymethylation in acid medium and treatment with 2,3-dichloro-5,6-dicyano-1,4-benzoquinone to yield 5,6,7,3′,4′-pentahydroxyflavone. Sinensetin was obtained by methylation of 5,6,7,3′,4′-pentahydroxyflavone using dimethyl sulphate and potassium carbonate.

### Pharmacological Activities and Mechanisms of Action

In this review, an updated overview of the pharmacological activities of sinensetin such as anticancer, anti-inflammatory, antioxidant, antimicrobial, anti-obesity, anti-dementia, vasorelaxant and antitrypanosomal activities and their underlying mechanisms of action are presented. The databases employed for data collection are mainly from Google Scholar, PubMed, Scopus and Science Direct. The keywords used during searching include ‘sinensetin’ AND ‘activity’ OR ‘antiproliferative’ OR ‘antiangiogenesis’ OR ‘antitrypanosomal’ OR ‘antimicrobial’ OR ‘antioxidant’ OR ‘antidementia’ OR ‘vasorelaxant’ OR ‘anti-inflammatory’ OR ‘anticancer’. The papers to be included need to fulfil the inclusion criteria as follows: original research papers related to any activity exhibited by sinensetin; sinensetin used can either be synthetic or an isolated natural compound but not in crude extract or mixture; published in English; experiments conducted are *in vivo* and/or *in vitro* and/or *ex vivo* assays. Most researchers used commercially available synthetic sinensetin to evaluate its pharmacological activities due to its limited supply from natural sources. The scientific information on sinensetin, sourced from natural sources and synthesis, are critically analysed to provide perspectives for future studies on the compound as a lead molecule for the development of new therapeutic agents. In addition, toxicological information have also been included to assess its safety before submission to clinical trials. Table 1 summarizes the various pharmacological activities of sinensetin such as anticancer (Choi et al., 2002, Choi et al., 2004; Mertens-Talcott et al., 2007; Ohtani et al., 2007; Du and Chen, 2010; Du and Chen, 2010; Tan et al., 2013; Zhang et al., 2014; Bai et al., 2019; Pereira et al., 2019); antiobesity (Sergeev et al., 2009; Kang et al., 2013, Kang et al., 2015), antioxidant (Lyckander and Malterud, 1996; Malterud and Rydland, 2000; Jeong et al., 2007; Yao et al., 2009); antidementia (Nagase et al., 2005; Kawahata et al., 2013; Kimura et al., 2013; Swaminathan et al., 2014; Youn et al., 2017), anti-inflammatory (Laavola et al., 2012; Shin et al., 2012; Chae et al., 2017), antiangiogenesis (Lam et al., 2012) and diuretic (Yuliana et al., 2009).

**TABLE 1:** Pharmacological activities of sinensetin.

Plant name/Source	Plant part	Assay type	Activity	Mechanism of action/conclusion	References
Synthetic: Shanghai Tauto Biotech Co.	–	*In vitro*	Antiangiogenesis	30 μM sinensetin exposed for 24 h obviously inhibits formation of angiogenic vessels, most potently inhibit formation of ISVs and DLAVs in Tg(*fli*1a: EGFP)γ1 besides suppressing VEGF-stimulated HUVEC proliferation dose-dependently with most potent activity (IC_50_ = 24 μM) after 48 h with *p* < 0.001 when compare with control (cells receiving 2.0 ng/ml) VEGF at 30, 60 and 100 µM	Lam et al. (2012)
*Citrus reticulata* Blanco	Peel	*In vitro*	Anticancer (Chemosensitizing)	Reverse the resistance to vincristine (IC_50_ = 1.14 µM) with highest CI (CI = 72.28), reversing P-glycoprotein-mediated MDR by increasing intracellular accumulation of drugs, chemosensitizing effect equipotent to that of verapamil (IC_50_ = 1.07 µM, CI = 47.57) or cyclosporine A (IC_50_ = 0.87 µM, CI = 16.70)	Choi et al. (2002)
–	–	*In vitro*	Anticancer (Chemosensitizing)	Reverse resistance to vincristine (IC_50_ > 3.2 µM) with CI > 125 in P-glycoprotein overexpressing AML-2/D100 using MTT assay as compared to Verapamil (IC_50_ > 0.4, CI = 152.5) µM as positive control	Choi et al. (2004)
Synthetic: Extrasynthese	–	*In vitro*	Anticancer (Chemosensitizing)	20 µM sinensetin did not change the uptake of [^3^H] vincristine into K562 cells but moderately increase uptake into K562/ADM cells in 2 h up to around 600% relative to that in the absence of sinensetin by inhibition of P-glycoprotein	Ohtani et al. (2007)
–	–	*In vitro*	Anticancer (Chemosensitizing)	Inhibition of P-glycoprotein. Sinensetin significantly reduced talinolol BL/AP transport in Caco-2 cell monolayer model with highest IC_50_, IC_50_ = 3.9 µM (95% CI: 1.8–8.4), I_max_ = 89% in concentration dependent manner. Values at 5, 10 and 50 μmol/L is significantly different (*p* ≤ 0.05) from the control. No significant influence reported on AP-BL transport as positive control, verapamil	Mertens-Talcott et al. (2007)
Synthetic: Extrasynthese	–	*In vitro*	Anticancer (Chemosensitizing)	Inhibition of ABCG2 which caused significant increase of mitroxantrone accumulation in HEK293/ABCG2 and HEK293/WT which are 181.7% ± 3.7 (*p* < 0.05) and 114.9% ± 2.6 (*p* < 0.05), respectively at 10 µM. Mitoxantrone accumulation in HEK293/ABCG2 and HEK293/WT by 5 µM Ko143 are 187.9% ± 34.4 and 118.2% ± 3.9, respectively with *p* < 0.05	Tan et al. (2013)
				Showed significant Sf9/ABCG 2 methotrexate membrane vesicular transport inhibition with percentage of 8.7% ± 0.3 (*p* < 0.05), 6.9% ± 0.8 (*p* < 0.05) at concentration of 5 and 50 µM as compared with untreated control (100%) and 1 µM Ko143 (4.0% ± 0.9, *p* < 0.05)	
Synthetic: Indofine	–	*In vitro*	Anticancer (Chemosensitizing)	IC_50_ = 3.2 µM in the presence of vincristine in AML-2/D100 with CI > 125 determined with MTT assay	Jeong et al. (2007)
Synthetic: J & K Scientific Co.	–	*In vitro*	Anticancer (Chemosensitizing)	37.8 µM sinensetin decreases MX-1 and MX-1/T cell viability as compared to control when exposed to 75 µM taxol	Bai et al. (2019)
		*In vitro*	Anticancer (Chemosensitizing)	100 µM sinensetin exhibits significant inhibition (79.68%) on P-gp tested on MDR1-MDCK II cells, remarkably increasing Papp(AP-BL)1.53 ± 0.28 (×10^–6^ cm/s) and decreasing Papp(BL-AP) 3.64 ± 0.45 (×10^–6^ cm/s) of 5 µg digoxin when compared to 5 µg PSC833 (96.28%, AP-BL = 2.27 ± 0.2 (×10^–6^ cm/s), BL-AP = 3.06 ± 1.32(×10^–6^ cm/s) and untreated control (AP-BL = 0.23 ± 0.09 (×10^–6^ cm/s, BL-AP = 21.4 ± 4.04 (×10^–6^ cm/s)	Bai et al. (2019)
		*In vivo*	Anticancer (Chemosensitizing)	Increase AUC_0-t_ 95.33 ± 6.29 h*ng/mL (*p* < 0.01) and C_max_ 25.85 ± 5.55 ng/ml (*p* < 0.05) relative to untreated control (AUC_0-t_ = 52.52 ± 3.78 h*ng/mL, C_max_ = 10.55 ± 1.45 ng/ml) while positive control verapamil resulted in AUC_0-t_ = 113.79 ± 17.63 h*ng/mL and C_max_ = 45.12 ± 10.81 ng/ml in male Sprague-Dawley rats	Bai et al. (2019)
*Citrus reticulata* Blanco	Peels 70% ethanol	*In vitro*	Anticancer (antiproliferative)	Resulted in IC_50_ values of 31.1 µM against H08910, 40.9 µM MCF-7, 47.5 µM HL-60 and 62.4 µM A590 cell lines using MTT assay, exhibited weakest antiproliferative activity in the entire cell lines	Du and Chen (2010)
*Citrus reticulata* Blanco	Flavedo 80% ethanol extract	*In vitro*	Anticancer (Antiproliferation)	Low activity (IC_50_ = 88.9 ± 32.3 μg/ml) against HepG2 among flavonoids tested within concentration range 0–100 μg/ml, no apparent effect below 100 μg/ml against MDA-MB-231 cells, more than 50% inhibitory rate for HL-60 and low inhibition effect on SKOV3 cell migration	Zhang et al. (2014)
Synthetic: Extrasynthese	–	*In vitro*	Anticancer (Antiproliferative)	Negligible antiproliferative effect in HT29 cell. Antiproliferative only in combination of nobiletin-sinensetin-tangeretin-scutellarian tetramethylether (IC_50_ = 1.24 ± 0.14 mg extract/mL) which equivalent concentration with that of orange peel extract (IC_50_ = 1.18 ± 0.07 mg extract/mL) at 72 h in 3D cell model of colorectal cancer composed of HT29 cell spheroids	Pereira et al. (2019)
		*In vitro*	Anticancer (Antiproliferative)	Sinensetin at 13.95–111.58 µM induced a higher antiproliferative response with 0.15–1.2 mg/ml 5-FU, exhibiting synergistic effects. The results are analysed and obtained by CompSyn software with combination index (CI) of 0.08–0.46 and dose reduction index for 5-FU range from 12.95–2.29	Pereira et al. (2019)
Synthetic: Funakoshi	–	*In vitro*	Anticancer (antiproliferative)	Showed IC_50_ values of more than 40 µM in A549, B16 melanoma 4A5, CCRF-HSB-2 and TGBC11TKB cell lines indicates weak antiproliferative activity	Kawaii et al. (1999)
Synthetic: Indofine	–	*In vitro*	Anticancer (antiproliferative)	CYP-1 mediated antiproliferative effect which inhibits strongly (*p* < 0.05) proliferation of MDA-MB-468 cells with IC_50_ = 0.2 ± 0.02 µM without affecting viability of MCF-10A with IC_50_ = 65 ± 5 µM for 96 h measured with MTT assay. Cotreatment with 1.5 µM acacetin reversed the inhibition (IC_50_ = 13.5 ± 3 µM)	Androutsopoulos et al. (2009)
Synthetic	–	*In vitro*	Anticancer (antiproliferative)	Antiproliferative with IC_50_ of 3.9 µM against MDA-MB-435 ER- (breast), 5.5 µM against MCF-7 ER+ (breast), 16.5 µM against DU-145 (prostate), 9.5 µM against HT-29(colon), 13.7 µM against DMS 114(lung) and 10.8 µM against SK-MEL5 (melanoma) measured by decrement of [^3^H] thymidine uptake	Manthey and Guthrie (2002)
*Citrus sinensis* (L.) Osbeck	Peel extract	*In vitro*	Anticancer (Apoptosis)	Increase in intracellular Ca^2+^, activation of apoptotic mechanism to cause inhibition of proliferation of MCF-7 in dose- and time-dependent manner within 7 days in dose ranged from 0–100 µM observed with propidium uptake resulted in weakest activity (IC_50_ > 50 µM) for induction of cell death, apoptosis, proliferation and inhibition of cell proliferation among PMF evaluated	Sergeev et al. (2006)
*C. sinensis* (L.) Osbeck	Acetone peel extract	*In vitro*	Anticancer	Moderately weak EBV-EA activation inhibition. Achieved 7.2% EBV-EA activation relative to TPA on 500 Raji cells at 1,000 mol ratio/TPA with high viability (70% at 1,000 mol ratio/TPA). Activation of 35.7% at 500 mol ratio/TPA, 65.0% at 100 mol ratio/TPA, 91.9% at 10 mol ratio/TPA were reported	Iwase et al. (2000)
*Orthosiphon spicatus* (Thunb.) Backer, Bakh.f. & Steenis	Plant extract	*In vitro*	Anticancer	Sinensetin show dose-dependent inhibitory effect towards Ehrlich acites tumor cells with IC_50_ = 30 μg/ml	Malterud et al. (1989)
Synthetic: Cayman chemical	–	*In vitro*	Anticancer	Caused significant (*p* < 0.001) cytotoxic effect to Jurkat (CC_50_ = 135.4 ± 12.3 μmol/L) and CCRF-CEM cells (CC_50_ = 198.3 ± 18.5 μmol/L) in a dose- and time-dependent manner within 48 h but exhibited moderate (*p* < 0.001) cytotoxicity (IC_50_ > 200 μmol/L) on BALB/c mouse primary T cell compared with 0.4% DMSO treated control group	Tan et al. (2019)
*Citrus reticulata* Blanco	Peel methanol extract	*In vitro*	Antidementia	Weakly stimulate ERK phosphorylation in PC12D cell with relative levels of p-ERK at 30 and 100 µM are low (both <4, not significant) when cells stimulated with sinensetin for 5 min vs. positive control, nobiletin (<8 and <12 respectively) and vehicle control, <0.3% DMSO (both <2)	Kimura et al. (2013)
*Citrus reticulata* Blanco	Peel methanol extract	*In vitro*	Antidementia	100 µM exposition for 5 h enhanced CRE-dependent transcription in PC12D cells (*p* < 0.01), for 48 h induced neurite outgrowth (*p* < 0.01) with 50 ng/ml NGF as control	Nagase et al. (2005)
*Citrus reticulata* Blanco	Peel extract	*In vitro*	Antidementia	Facilitate CRE-mediated transcription linked to upstream cAMP/PKA/ERK/CREB pathway in neuron with most potent activity in facilitating transcription at 30 µM on hippocampal neuron in primary culture from embryos of an 18-days pregnant Sprague-Dawley rat (*p* < 0.001 vs. 0.1% DMSO, *p* < 0.01 vs. nobiletin)	Kawahata et al. (2013)
Synthetic: Sigma-Aldrich	–	*In vitro*	Antidementia	Inhibit BACE1 with IC_50_ value of 6.3 × 10^–5^ M to lower Aβ generation. Inhibition in a dose-dependent (*p* < 0.001) and non-competitive manner with K_i_ value of 3.8 × 10^–5^ M. No statistically significant inhibition against TACE and other serine proteases (trypsin, chymotrypsin, and elastase), inhibition is specific	Youn et al. (2017)
Synthetic	–	*In vitro*	Antidementia	M1 muscarinic receptor binding depicted weak activity (IC_50_ > 10,000 µM) with absence of Ki value to displace [3H] N-methylscopolamine using cloned human M1 mAChRs as compared to acetylcholine (IC_50_ = 142 ± 12 μM, K_i_ = 59 ± 5 µM) and pilocarpine (IC_50_ = 6.5 ± 0.1 µM, K_i_ = 2.7 ± 0.1 µM)	Swaminathan et al. (2014)
Synthetic: ChromaDex	–	*In vitro*	Anti-inflammatory	Pre-treating LPS-stimulated RAW 264.7 macrophage cell for 1 h dose-dependently inhibits secretion of NO and protein expression of iNOS and COX-2. 50 μM sinensetin for 1 h attenuates level of IL-1β, IL-6 and TNF-α mRNA (*p* < 0.005), significantly delay LPS-induced IĸB-α disappearance and enhanced reappearance, decreases nuclear level of p65 in cells within 50 min	Shin et al. (2012)
Synthetic: ChromaDex	–	*In vitro*	Anti-inflammatory	Inhibits IL-6 in human mast cell-1 via STAT3 and NF-ĸB pathways. Inhibits PMA plus A23187 induced IL-6 production (*p* < 0.05 at 0.8 and 4 μM, *p* < 0.005 at 20 μM) so as IL-4, IL-5 and IL-8 mRNA expression (*p* < 0.05 at 20 μM), inhibit STAT-3 phosphorylation and inhibits NF-ĸB activation by inhibiting IĸB-α degradation and p65 translocation, all in dose-dependent manner	Chae et al. (2017)
Synthetic: Department of Citrus, State of Florida, Lakeland, FL	–	*In vitro*	Anti-inflammatory	Lacked of activity against f-MetLeuPhe-induced human neutrophil beta-glucoronidase release (IC_50_ > 50 μM). Inhibited both antigen-(IC_50_ = 44 μM) and TPA-stimulated (IC_50_ = 26 μM) human basophil histamine release moderately but did not affect ionophore-induced release (IC_50_ > 50 μM)	Middleton et al. (1987)
Synthetic: Extrasynthese	–	*In vitro*	Anti-inflammatory	Inhibit iNOS expression at 5 μM (*p* < 0.05) and 50 μM (*p* < 0.01), inhibit NO (IC_50_ = 9.2 μM) and TNF-α (IC_50_ = 2.7 μM) production in J774 murine macrophages. 10 μM inhibit LPS-induced iNOS mRNA expression by more than 50% (*p* < 0.01). 50 μM inhibited COX-2 expression (*p* < 0.01) and PGE_2_ (IC_50_ = 2.7 μM) in cells stimulated with LPS in a dose-dependent manner (*p* < 0.01) at 0–100 μM range. 10 µM Near full inhibition of LPS-induced activation of transcription factor STAT1α comparable to 10 µM dexamethasone used as control (*p* < 0.05)	Laavola et al. (2012)
				No inhibition of NF-ĸB p65 nuclear translocation at 10 µM	
Synthetic: Extrasynthese	–	*In vivo*	Anti-inflammatory	Pre-treat 50 mg/kg i.p 2 h prior to carrageenan injection inhibited carrageenan-induced paw inflammation by 50% in C57BL/6 mice (*p* < 0.001) while dexamethasone 2 mg/kg inhibited by 84%	Laavola et al. (2012)
Synthetic: Chromadex	–	*In vitro*	Antimicrobial	More effective antagonist against AI-2-mediated bioluminescence in reporter strains *V. harveyi* MM32 than against *V. harveyi* BB886 (HAI-1- mediated) and depicted strong inhibition of *E. coli* O157:H7 and *V. harveyi* biofilm formation	Vikram et al. (2010)
Synthetic	–	*In vitro*	Antimicrobial	100 μM significantly inhibit *E.coli* O157:H7 motility halos at 24 h (*p* < 0.01) identified by zone diameter at 12 and 24 h	Vikram et al. (2013)
*Citrus sinensis* (L.) Osberk	extract	*In vitro*	Antiobesity	Weakly induce apoptosis (EC_50_ > 100 μM, 46.8 and 56.1 μM at day 1, 3 and 6) in mature mouse 3T3-L1 adipocytes via activation of Ca^2+^-dependent calpain and Ca^2+^/calpain-dependent caspase-12 in 3T3-L1 adipocytes. Treatment of sinensetin resulted in sustained increase in the basal level of intracellular Ca^2+^, time- and concentration-dependently, with EC_50_ value of more than 100 µM for day1 and day 6 to increase [Ca^2+^]. Significant caplain and caspase-12 activities (*p* < 0.05) between day 3 and day 6 of the treatment in mature adipocytes treated with 100 µM sinensetin	Sergeev et al. (2009)
*Citrus reticulata* Blanco	Peel aqueous extract	*In vitro*	Antiobesity	Decreases expression of SREBP-1c in 3T3-L1, increases phosphorylation of PKA and hormone-sensitive lipase at 40 μM, inhibits insulin-stimulated glucose uptake (*p* < 0.05 at 40 µM) by decreasing phosphorylation of insulin receptor substrate and Akt. Increases phosphorylation of AMPK and acetyl-CoA carboxylases, upregulate mRNA expression of CPT-1a (*p* < 0.05 at 40 µM) with DMSO treated cells as control	Kang et al. (2013)
Synthetic: ChromaDex	–	*In vitro*	Antiobesity	Promote adipogenesis in preadipocytes and lipolysis in mature adipocytes via cAMP pathway. Upregulate expression of PPAR-γ, C/EBP *α* and SREBP-1c, potentiated expression of C/EBP *β* and activation of cAMP-responsive element-binding protein, enhanced activation of PKA and increase intracellular cAMP in 3T3-L1 cells	Kang et al. (2015)
				Significantly (*p* < 0.05) increased lipolysis at 24 and 48 h after treatment measured with glycerol level associated with increment of cAMP at 0–40 µM in mature 3T3-L1 adipocytes	
*Citrus sinensis* (L.) Osberk	Peel ethanol extract	*In vitro*	Antioxidant	Moderate antioxidant effect in dose dependent manner between 3.5–70 μg/ml on superoxide anion with IC_50_ values of 35.52 ± 0.15 μg/ml	Yao et al. (2009)
*Citrus sinensis* (L.) Osbeck	Peel methanol extract	*In vitro*	Antioxidant	Inhibit 15-lipooxygenase less actively (IC_50_ = 74 ± 7 µM) as compared to positive control, quercetin (IC_50_ = 68 ± 5 µM)	Malterud and Rydland (2000)
*Orthosiphon spicatus* (Thunb.) Backer, Bakh.f. & Steenis	Ethyl acetate extract	*In vitro*	Antioxidant	Moderately active protective activity towards air-induced inactivation of soybean 15-lipooxygenase is obtained as I_50_ = 21.0 ± 2 µM and IC_50_ = 114 ± 5 µM. Not efficient scavenger with 0.6 ± 0.1% DPPH radical scavenged at 167 μM, no significant protection for sulfhydryl group at 10–160 μM, dose-dependent stabilizer of enzyme with poor activity	Lyckander and Malterud (1996)
Synthetic: Indofine	–	*In vitro*	Antioxidant	100 µM showed low antioxidant activity in DPPH assay (10.1%, *p* < 0.05) comparable to NAC (13.2%) or PDTC (15.2%), comparable activity to Vitamin E (33.4%) in DCFH assay (26.2%, *p* < 0.05) in AML-2/DX100 cells with IC_50_ = 3.2 µM	Jeong et al. (2007)
Cultivar Setoka Synthetic: Funakoshi	Peel ethnolic extract	*In vitro*	Antitrypanosomal	Extracted “Setoka” sinensetin and reagent grade sinensetin moderately inhibit proliferation of *Trypanosoma brucei* with IC_50_ = 4.8 ± 0.4 and IC_50_ = 4.1 ± 0.04 μg/ml, respectively	Nakanishi et al. (2019)
*Orthosiphon aristatus* (Blume) Miq.	80% methanol extract	*In vitro*	Diuretic	A1-R-active ligand with pK_i_ value and hill slope of 5.5 and 0.9 respectively comparable to luteolin (pK_i_ = 5.4 ± 0.2, hill slope = 1.3) and quercetin (pK_i_ = 5.8 ± 0.1, hill slope = 1.1)	Yuliana et al. (2009)
Synthetic: Extrasynthese	–	*In vitro*	Lipid lowering	Weak suppression of apoB secretion. Had weak effect on CH and TG syntheses (data not shown) against HepG2 cells. IC_50_ > 100 µM on inhibition of both albumin and apoB secretion by HepG2 cells incubated with sinensetin for 24 h at 0–100 µM determined by ELISA	Lin et al. (2011)
Synthetic: Sigma Aldrich	–	*In vitro*	Vasorelaxant	0.03–2.11 μM sinensetin caused concentration-dependent vasorelaxation of PE-contracted endothelium-intact aortic rings (pD2 = 6.97 ± 0.09 and *E*_max_ = 108 ± 9.83%) via NO/sGC/cGMP and indomenthacin pathways, potassium and calcium channels, and muscarinic and beta-adrenergic receptors	Yam et al. (2018)

### Anticancer Activity

The search for new anticancer drugs with high therapeutic index has led to great extent of anticancer research on plant-derived natural products such as flavonoids. Sinensetin is one of the polymethoxylated flavonoids that has been intensively studied for its anticancer activity. Chemotherapy remains as a standard in the treatment and management of cancer (Qiu et al., 2019). However, the major obstacle to successful chemotherapy is the development of multidrug resistance (MDR) by tumor cells after long term administration of chemotherapy drugs (Choi et al., 2002; Qiu et al., 2019). Prominent group of ATP-binding cassette (ABC) transporters such as P-glycoprotein (P-gp), Multiresistance Protein (MRP) and ATP Binding Cassette Subfamily G Member or also known as breast cancer resistance protein (ABCG2 or BCRP) have been characterized in relation to MDR, limiting the exposure to anticancer drugs (Choi and Yu, 2014; Wilkens, 2015; Waghray and Zhang, 2018). Sinensetin has been intensively investigated for its anticancer activities and its mechanisms of anticancer activities involving interaction with P-gp, breast cancer resistance protein (ABCG2/BCRP), antiproliferation, and antiangiogenesis are critically discussed.

### Interaction of Sinensetin With P-Glycoprotein

Sinensetin has been reported to inhibit P-glycoprotein (P-gp) in several studies. Elevated expression of P-glycoprotein (P-gp) due to regulation of human gene expression of MDR1 is a significant cause for tumor multidrug resistance (MDR) (Qiu et al., 2019). Overexpression of P-gp has been linked to reduced chemotherapeutic responses and poor clinical outcomes in various cancer types (Waghray and Zhang, 2018). Therefore, P-gp inhibition is nominated as an effective strategy to reverse MDR (Qiu et al., 2019). Sinensetin, was found to significantly inhibit P-gp in a few cell types such as MDR1-MDCK II cells, Caco 2 cells and K562/ADM cells (Mertens-Talcott et al., 2007; Ohtani et al., 2007; Bai et al., 2019). Subsequently, sinensetin was reported to induce antiproliferation response synergistically with 5-fluorouracil in HT29 cell (Pereira et al., 2019). These findings revealed the potential of sinensetin as a new flavonoid chemosensitizer. Chemosensitizers are in need so that MDR cells can be sensitized to anticancer drugs when treated simultaneously (Choi et al., 2002). Calcium channel blocker, verapamil and various drugs such as phenothiazine, quinidine, cyclosporine A, valspodar (PSC 833) and much more had been reported to be potential drugs to overcome MDR (Wang et al., 1995; Fromm et al., 1999; Tidefelt et al., 2000; Summers et al., 2004; Wesolowska et al., 2009). While many compounds able to inhibit P-gp function have been identified, no broadly applicable inhibitor is in use yet. This is due to poor potency, significant side effects, unsatisfactory toxicity and low selectivity of the compounds (Wilkens, 2015). Potent and nontoxic P-gp inhibitors are needed to increase drug accumulation and reversal of drug resistance (Leonard et al., 2003). Choi et al. (2002) reported that sinensetin exhibited the most potent chemosensitizing effect among all tested flavonoids with an IC_50_ value (1.14 µM) comparable to that of verapamil (IC_50_ = 1.07 µM) and cyclosporine (IC_50_ = 0.87 µM), with the presence of vincristine in P-gp overexpressing AML-2/D100 cells. Sinensetin inhibitory activity was observed in the presence of vincristine which is also a good substrate of P-gp. The inhibitory effect of sinensetin on P-gp was well-supported by other studies (Mertens-Talcott et al., 2007; Bai et al., 2019; Danisman Kalindemirtas et al., 2019). However, there was no chemosensitizing activity of sinensetin against MRP-overexpressing cancer cells AML-2/DX100 (Choi et al., 2002). In a further screening of flavonoid sensitizer conducted by Choi et al. (2004), sinensetin was reported to exhibit IC_50_ value of 3.2 µM (chemosensitizing index, CI = 125).

Aside from chemosensitizing, sinensetin aided in the reversal of MDR phenotype in aspects such as decreased accumulation of the drug and enhanced efflux of anticancer drugs. As previously reported by Choi et al. (2002), sinensetin significantly (*p* < 0.05) increased the accumulation of rhodamine-123, a drug transported by P-gp in AML-2/D100 at a concentration of 20 µM. However, the dose accumulation determined by flow cytometry of sinensetin was 4- and 16-folds lower than those of verapamil and cyclosporine A, when concentrations were compared to induce the same effect. The difference thought may be due to different incubation times or other cellular effects of sinensetin than P-gp inhibition (Choi et al., 2002). Sinensetin increased digoxin uptake in rats with significant increment relative to control in AUC_0-t_ and C_max_ although weaker than verapamil as a positive control. The effects of several flavonoids including sinensetin on the pharmacokinetics of digoxin in rats after administration of 0.25 mg/kg digoxin were evaluated. Results disclosed significant increment in AUC_0-t_ 95.33 (*p* < 0.01) and C_max_ 25.85 (*p* < 0.05) caused by sinensetin. In addition, Vd/F and Cl/F of digoxin were also decreased after pre-administration with sinensetin with 11.6 (*p* < 0.05) and 2.48 (*p* < 0.01), respectively (Bai et al., 2019). These findings on sinensetin were consistent with increment of rhodamine-123 accumulation in AML-2/D100 (Choi et al., 2002), reduced talinolol transport by P-gp from basolateral to apical (BL/AP) side in Caco-2 colon carcinoma cell monolayer model (Mertens-Talcott et al., 2007) and increased uptake of [^3^H] vincristine into adriamycin-resistant human myelogenous leukemia (K562/ADM) cells (Ohtani et al., 2007).

Sinensetin also decreased P-gp levels even at the maximum non-cytotoxic concentration unlike verapamil, cyclosporine A and nefedipine which increased P-gp expression with an increase in MDR1 mRNA level (Choi et al., 2002). Maximum non cytotoxic concentration (more than 90% of control survival) of sinensetin, 25 µM was administered to AML-2/D100 for 72 h and appeared to decrease P-gp levels in Western blot analysis. Meanwhile, verapamil and cyclosporine A showed an increase in P-gp levels. This may suggest long term use of sinensetin as a better chemosensitizing agent than verapamil and cyclosporine A without concern about P-gp activation. Modulators blocked the substrate transportation by binding competitively and noncompetitively with P-gp. Apart from that, cyclosporin A and verapamil partially inhibited photolabeling of P-gp by [3^H^] azidopine but not by sinensetin at the same excess molar concentrations of 100 µM. This result may suggest that sinensetin did not interact directly with P-gp at the azidopine-binding site. Collectively, sinensetin was believed to have significant advantages of having a high therapeutic index or chemosensitizing index (CI), of being a non-transportable inhibitor and having no induction of P-gp in long term use.

#### Interaction of Sinensetin With Breast Cancer Resistance Protein

Sinensetin has been shown to inhibit the efflux transporter, ATP Binding Cassette Subfamily G Member or also known as breast cancer resistance protein (ABCG2 or BCRP), which is one of the players taking part in multidrug resistance same as P-gp (Mo and Zhang, 2012). Overexpression of ABCG2 has been studied and correlated to MDR phenotypes of numerous cancer cell lines derived from tumor types such as SN-38-selected human small cell lung cancer cells PC-6/SN2-5, mitoxantrone-selected human gastric carcinoma cell line EPG85-257RNOV, gefitinib-resistant non-small cell lung cancer (NSCLC) cells, epirubicin-resistant human hepatocyte carcinoma cells HLE-EPI, topotecan and doxorubicin-selected human multiple myeloma cells (Mo and Zhang, 2012). ABCG2 contributed to cancer progression due to its active efflux of chemotherapeutic agents out of neoplastic cells (Natarajan et al., 2012). The inhibiting properties of sinensetin against the efflux transporter ABCG2 was determined by *in vitro* mitoxantrone accumulation assay using wild type HEK293 (human embryonic kidney), HEK293/WT and ABCG2-overexpressing HEK293 cell line (HEK293/ABCG2) and sf9 (*Spodoptera frugiperda*) insect cell membrane vesicles overexpressing ABCG2 for the cell-based and the membrane-based methods, respectively. The results showed that 10 µM sinensetin significantly (*p* < 0.05) enhanced mitoxantrone accumulation in both HEK293/ABCG2 (181.7%) and HEK 293/WT cells (114.9%). However, mitroxantrone accumulation increment in HEK 293/WT cells has no significant difference from 5 µM Ko143 treatment effect (118.2%, *p* < 0.05). Sinensetin restricted transport of methotrexate into Sf9/ABCG2 to 8.7% (*p* < 0.05) at 5 μM and 6.9% (*p* < 0.05) at 50 μM, which were comparable to 1 µM Ko143 (4.0%) (Tan et al., 2013). The identified ABCG2 modulating ability of sinensetin may provide a framework for further investigation to identify potential MDR agents from phytochemicals.

#### Antiproliferation Effect of Sinensetin

Cancer stem cell (CSC), considered as one of the main causes of resistance to chemotherapeutic drug, is responsible for tumor initiation, relapse, invasion and metastases (Ishimoto et al., 2015; Otoukesh et al., 2018). MicroRNAs can participate in modulating multiple genes and CSCs functions (Otoukesh et al., 2018). Several miRNAs were found to target mRNA of epithelial-mesenchymal transition (EMT) such as ZEB1, ZEB2, or SNAI1 (Vu and Datta, 2017). Thus, it can also be one of the ways to target CSC. Synergistic interaction of sinensetin with 5-fluorouracil besides modulation of EMT genes has been reported (Pereira et al., 2019). EMT genes were modulated by sinensetin such that tumor suppressor gene, CDH1 was increased while ZEB1 and SNAI1 were reduced. Sinensetin alone did not present antiproliferative effect. However, nobiletin-sinensetin-tangeretin-scutellarian tetramethylether (N-S-T-SC), mixture of four polymethoxylated flavones (PMF) with concentration equivalent to that of orange peel extract (OPE) presented similar response as OPE. In terms of antiproliferation, expression of cell cycle marker, CDKN1A (coding for p21) and CCNA2 (coding for cyclin A2) genes and the apoptotic marker BICR5 (encoding baculoviral IAP repeat-containing protein 5) were also evaluated with sinensetin. No significant activity was observed on lowering expression of BICR5, presented lower numbers of caspase-3 active cells to induce apoptosis as BICR5 prevented apoptosis by caspase-3 inhibition. Besides, overexpression of CDKN1A might lead to G1/S cell cycle arrest while reduced CCNA2 was proposed to arrest G2/M cell cycle. However, the effect of sinensetin was negligible in the aspect of antiproliferation in HT29 cell and this was supported by expression data of BICR5*,* CDKN1A and CCNA2. Expression of cancer stemness markers (PROM1 and LGR5)*,* EMT genes (SNAI1, ZEB1 gene and CDH1) and percentage of ALDH + cells were evaluated to assess the effect of sinensetin in modulating cancer stemness and self-renewal. Sinensetin at 46.62 µM, reduced LGR5 (*p* ≤ 0.001), decreased expression of mesenchymal markers, SNAI1 (*p* ≤ 0.001) and ZEB1 (*p* ≤ 0.001) and increased CDH1 (*p* ≤ 0.0001), the results were expressed relative to untreated control (Pereira et al., 2019).

According to Androutsopoulos et al. (2009), sinensetin possessed CYP-1 mediated antiproliferative activity in MDA-MB-468 breast adenocarcinoma cell line. Sinensetin strongly inhibited (*p* < 0.05) *in vitro* proliferation of MDA-MB-468 cells with IC_50_ = 0.2 µM without affecting the viability of normal breast cell line MCF-10A with IC_50_ = 65 µM for 96 h measured with MTT assay. Co-treatment of sinensetin with 1.5 µM acacetin reversed the inhibition (IC_50_ = 13.5 µM), confirming the involvement of CYP1 as acacetin is a CYP1 family enzyme inhibitor. Sinensetin was metabolised by CYP1 enzymes and produced various metabolites which were unidentified by HPLC analysis. The metabolites were suggested to be involved in its antiproliferative activity (Androutsopoulos et al., 2009). In the study conducted by Iwase et al. (2000), sinensetin exhibited 7.2% relative to control (100%) on Epstein-Barr virus (EBV-EA) activation without causing cytotoxic effect on Raji cells with 70% viability. Evaluation of EBV-EA inhibition is now applied to identify possible anti-tumor agents (Kapadia et al., 2000). The inhibition effect was moderately weak as the reported range of all the compounds tested was 0–12.7% of activation. Manthey and Guthrie (2002) studied the effects of several classes of phenols, 40 natural and synthetic flavonoids on six cancer cell lines including MDA-MB-435 human breast cancer cell, MCF-7 ER + human breast cancer cells, DU-145 androgen receptor-negative human prostate cancer cells, HT-29 human colon cancer cells, DMS-114 human lung cancer cells and SK-MEL5 human melanoma cells. Sinensetin showed a strong antiproliferative activity against MDA-MB-435 ER-and MCF-7 ER+, moderate against HT-29, DMS 114 and SK-MEL5 and weakest against DU-145, measured by decrement of [^3^H] thymidine uptake in treated cells. In addition, it is interesting to find that the activity of 5-desmethyl sinensetin was significantly higher than sinensetin with lower IC_50_ values against all cell lines (Manthey and Guthrie, 2002). In another study, sinensetin isolated from the flavedo of *C. reticulata* exhibited more than 50% inhibitory rate on promyelocytic leukemia HL-60 cells. Activity against Hep G2 liver cells although reported low s, it was the first time reported for antiproliferative effect on the growth of liver cancer (Zhang et al., 2014).

Sinensetin was found to significantly impede Jurkat cell proliferation and additionally triggered apoptosis and autophagy (Tan et al., 2019). Human T-cell acute lymphoblastic leukemia, CCRF-CEM, non-Hodgkin’s lymphoma cell line, Jurkat cell and mouse primary T-cells isolated and purified from six week-old male BALB/c mice were used in this study. Sinensetin inhibited cell viability in different strength at concentration ranged 1–100 μmol/L for 24 or 48 h compared with 0.4% DMSO-treated control group dose- and time-dependently. It caused significant (*p* < 0.001) cytotoxic effects to Jurkat and CCRF- CEM cells at concentrations of 25–100 µmol but moderate (*p* < 0.01) toxicity to mouse primary T-cells at a dose range of 100–200 μM at 24 and 48 h. Based on cell viability results evaluated by CCK-8 assays, IC_50_ values at 48 h were 135.4, 198.3 and >200 μmol/L for Jurkat, CCRF- CEM cells and mouse primary T-cells, respectively. Sinensetin induced sub-G1 population, an indicator of apoptotic death significantly (*p* < 0.001) when Jurkat cell treated with 50 and 100 µM for 24 and 48 h, suggesting cell-cycle arrest was involved in the growth inhibition. Considerable increase (*p* < 0.001) in total Annexin V+ apoptotic cells in Jurkat cells treated with sinensetin was observed. Membrane potential and protein expression of cleaved poly (ADP-ribose) polymerase (cleaved-PARP), caspase-3, caspase-8 and caspase-9 were significantly increased in Jurkat cell treated with sinensetin. The results suggested that sinensetin provoked caspase-dependent apoptosis. Acid vesicular organelles (AVOs) were used to detect autophagy. Cells treated also revealed an exhibition of more AVOs (*p* < 0.001) which increased with time and concentration. In the same study, sinensetin downregulated phosphorylation of Akt and mTOR dose-dependently. Inhibition of those two tremendously led Jurkat cells to be more sensitive to cytotoxicity effect of sinensetin in Jurkat cells, suggest that co-treatment of sinensetin with Akt and mTOR inhibitor synergistically blocked Akt/mTOR signaling pathway (Tan et al., 2019).

#### Antiangiogenesis Effect of Sinensetin

Growing evidences of the correlation between angiogenesis and chronic inflammation urge the search for effective natural occurring antiangiogenesis agents. In the study by Lam et al. (2012), sinensetin was reported as having the most potent antiangiogenesis activity with the lowest toxicity among seven polymethoxylated flavonoids. Sinensetin inhibited angiogenesis by inducing cell cycle arrest in the G0/G1 phase in human umbilical vein endothelial cell (HUVEC) culture and downregulating the mRNA expressions of angiogenesis genes flt 1, kdrl, and hras in zebrafish (*Danio rerio*). Sinensetin at 30 µM demonstrated the most potent inhibition on formation of intersegmental vessels (ISVs) and dorsal longitudinal anastomotic vessels (DLAVs) when transgenic zebrafish embryos, Tg (fli1a: EGFP) γ1 incubated for 24 h compared to hesperidin, nobiletin, scutellarein and scutellarein tetramethylether (STE). At 3 μM sinensetin affected the formation of ISVs with mild antiangiogenesis activity while 30 μM almost completely arrested the growth of ISVs. Sinensetin has the most potent inhibitory activity (IC_50_ = 24 µM) and significant dose-dependent suppression of VEGF-induced HUVEC proliferation after 48 h at a concentration range of 0–100 µM with cells receiving VEGF (20 ng/ml) served as a control. Inhibition of HUVEC proliferation might be due to cell cycle arrest in the G0/G1 phase. The expression of flt1, hras, and kdrl mRNA were downregulated by 0.56, 0.44, and 0.52-fold, respectively, after treatment with 30 μM sinensetin. Meanwhile, after treatment with 10 and 30 μM sinensetin the expression of vegfaa mRNA was significantly upregulated by about 1.70 fold as compared to control.

### Anti-inflammatory Activity

Previously, many flavonoids had been found to be affecting the function of cells such as mast cell, basophils, neutrophils and platelets to modulate allergy and inflammatory responses (Middleton et al., 1987). Activated macrophage induces pro-inflammatory mediators such as PGE2, NO, IL-1β, IL-6 and TNF-α produced by inflammatory enzymes iNOS and COX-2 to cause damage to pathogen and host cells (Laavola et al., 2012; Shin et al., 2012). Middleton et al. (1987) reported sinensetin showed moderate activity on the release of basophil histamine but not neutrophil beta-glucuronidase. Basophil histamine release stimulated by antigen, tetradecanoyl phorbol acetate (TPA) and calcium ionophore, A 23,187 were by distinct immunologic, receptor-directed and non-receptor directed pathways, respectively. Sinensetin inhibited both antigen- and TPA-stimulated histamine release with IC_50_ values of 44 and 26 μM, respectively (Middleton et al., 1987).

Similar activities were displayed in different mechanisms and cells by other workers (Laavola et al., 2012; Shin et al., 2012; Chae et al., 2017). LPS and TNF-α are stimuli that activate macrophages during an inflammatory response. The anti-inflammatory activity of sinensetin was studied by using LPS-stimulated RAW 264.7 cells. LPS produces inflammatory response by activating MAPK and NF-ĸB pathways which involve degradation of inhibitor ĸB (iĸB). Sinensetin inhibited NF-ĸB activation by inhibiting degradation or stabilizing of IĸB-α, suppressing excessive inflammation with no effect on MAPK phosphorylation (Shin et al., 2012; Chae et al., 2017). Activated NF-ĸB can be terminated by the export of nuclear NF-ĸB and degradation of p65 in the nucleus. Pre-treating LPS-stimulated RAW 264.7 macrophage cell for 1 h inhibited secretion of NO and protein expression of iNOS and COX-2 dose-dependently. Sinensetin at 50 μM attenuated the level of pro-inflammatory cytokines, IL-1β, IL-6 and TNF-α mRNA (*p* < 0.005). Significantly delayed LPS-induced IĸB-α disappearance and enhanced reappearance were also reported, together with the finding of decreased nuclear level of p65 in cells within 50 min (Shin et al., 2012). Similar activity was displayed by dose-dependent inhibition of p65 translocation in human mast cell-1 (HMC-1) (Chae et al., 2017).

Laavola et al. (2012) further revealed sinensetin inhibited the production of prostaglandin E2 (PGE2) with IC_50_ value of 2.7 µM in a dose-dependent manner besides providing evidence to support the findings of Shin et al. (2012) on iNOS and COX-2 expression and NO production inhibition. In the carrageenan-induced paw inflammation test, sinensetin (50 mg/kg) treatment via intraperitoneal route was reported to inhibit carrageenan-induced paw inflammation in C57BL/6 mice (Laavola et al., 2012). The effect was comparable to 2 mg/kg dexamethasone which inhibited paw edema response by 84%. Chae et al. (2017) investigated human mast cell-1 (HMC1) mediated inflammatory mechanism. It was found that sinensetin inhibited PMA plus A23187-induced IL-6 production in a dose-dependent manner as well as IL-4, IL-5 and IL-8 mRNA expressions. Elevated level of IL-6 in stimulated mast cell was lowered by treatment with sinensetin, at a concentration range of 0.8–0.20 µM. Treatment of PMA plus A23187 induced IL-6 production and suppression of increased mRNA expression with the treatment of sinensetin was assayed by RT-PCR. Increment of IL-4, IL-5 and IL-8 mRNA expression in 6 h stimulated HMC-1 were significantly (*p* < 0.05) decreased with pretreatment of 20 µM sinensetin for 0.5 h (Chae et al., 2017). Sinensetin also inhibited signal transducer and activation of transcription 3 (STAT3) phosphorylation in PMA plus A23187-stimulated HMC-1. Activated STAT3 up-regulated the expression of inflammation mediators and induced inflammation-relevant gene expression. The inhibition of sinensetin was in a dose-dependent manner with HMC-1 pretreated with sinensetin for 0.5 h and stimulated for 0.5 h to detect phosphorylated STAT3 (Chae et al., 2017). Overall, these findings proposed sinensetin involvement in mast cell-mediated inflammatory responses, providing a clue for further findings on anti-inflammatory mechanisms in the future.

### Antioxidant Activity

Inhibition of 15-lipooxygenase is of interest for researchers as it was proposed to have a role in oxidation of low density lipoprotein (LDL) which believed to be a significant step leading to atherosclerotic lesions. Air may induce inactivation of 15-lipooxygenase by loss of sulfhydryl groups through oxidation. Sinensetin was a moderate inhibitor of air-induced inactivation of soybean 15-lipooxygenase with an IC_50_ value of 114 µM besides acting as a poor stabilizer of enzyme which did not possess significant protection for sulfhydryl group at concentrations up to 160 μM, as reported by Lyckander and Malterud (1996). In a study by Malterud and Ryland (2000), sinensetin inhibited 15-lipooxyygenase with an IC_50_ value of 74.0 µM which was higher than that of the positive control, quercetin (IC_50_ = 68 ± 5 µM). Free radical scavenging activity of sinensetin was assayed by using diphenylpicryhydrayzyl (DPPH) and dichlorofluorescin (DCFH). Sinensetin showed low antioxidant activity in DPPH assay (Lyckander and Malterud, 1996), employing AML2/DX100 cells which possessed supersensitivity towards hydrogen peroxide treatment due to down-regulated catalase expression comparable to positive control antioxidants, *N*-acetyl-l-cysteine (NAC) or pyrrolidine dithiocarbamate (PDTC). However, comparable antioxidant activity to vitamin E (33.4%) was observed in DCFH fluorescence-scavenging assay (26.2%, *p* < 0.05). Besides, Yao et al. (2009) revealed antioxidant effect of sinensetin in a dose dependent manner between 3.5 and 70 μg/ml on superoxide anion with an IC_50_ value of 35.52 μg/ml.

### Effects on Lipolysis Metabolism

Previous study by Sergeev et al. (2006) indicated that sinensetin induced apoptosis *via* increasing basal intracellular Ca^2+^ level and activation of Ca^2+^ mediated, caplain-dependent caspase-12. There are different *in vitro* experiments conducted on 3T3-L1 preadipocyte cells to study the effects and mechanisms of sinensetin on lipid metabolism. The adipocytes generated exhibited most of the key features of adipocytes *in vivo* for the 3T3-L1 cells. Induction of adipocyte apoptosis to reduce body fat may result in long-lasting weight loss, as compared with after caloric restriction as well as bariatric surgery. Sergeev et al. (2006) undertaken a study to determine whether polymethoxyflavones (PMFs) including 5-hydroxy-3,6,7,8,3′,4′-hexamethoxyflavone, 3,5,6,7,8,3′,4′-heptamethoxyflavone, 5,6,7,3′,4′-pentamethoxyflavone and 3′-hydroxy-5,6,7,4′-tetramethoxyflavone could induce Ca^2+^-mediated apoptosis in mature adipocytes. All the PMFs increased basal levels of intracellular Ca^2+^ and induced apoptosis in dose and time-dependent manner in mature 3T3-L1 adipocytes treated with PMFs of concentration range between 3.125 and 100 µM for 1, 3, or 6 days. Sinensetin increased intracellular Ca^2+^ with EC_50_ of >100 μM, 45.9 µM, >100 µM for 1, 3 and 6 days, respectively. As for the induction of apoptosis, EC_50_ values of all the PMFs including sinensetin were generally the same range as those for inducing intracellular Ca^2+^. However, the effective concentration for inducing apoptosis was lower than those for inducing intracellular Ca^2+^ at day 6 of treatment with an EC_50_ value of 56.1 µM. Cellular apoptotic changes were also shown when treated at 50 µM for 3 and 6 days labelled with Annexin V-Alexa Fluor 488. Subsequently, it was reported that the sustained increase of intracellular Ca^2+^ level was accompanied by activation of caplain and caspase-12 activities measured with specific fluorogenic peptide substrates when treated with sinensetin (*p* < 0.05 for day 3). From this experiment, it was found that sinensetin induced apoptosis *via* increasing basal intracellular Ca^2+^ level and activation of Ca^2+^ mediated, caplain-dependent caspase-12.

Besides, adipose tissue lipolysis actually controls the quantity of triglycerides stored in fat depots and in determining plasma free fatty acid levels (Kang et al., 2013). Effects and lipid metabolism of sinensetin isolated from PMF-rich *C. reticulata* peel was studied and the mechanism or pathway was elucidated in mature 3T3-L1 adipocytes. A decrease in the level of sterol regulatory element-binding protein (SREBP) 1c expression was revealed when treated with 40 µM of sinensetin. Sinensetin increased glycerol released into culture supernatants and stimulated phosphorylation of protein kinase A (PKA) and hormone-sensitive lipase (HSL), suggesting that sinensetin enhanced lipolysis by cAMP-dependent PKA. Glucose uptake and phosphorylation of Akt and IRS both were inhibited when treated by sinensetin. The results proposed that by reduction of phospho-insulin receptor substrate (IRS) and phosphor-Akt, sinensetin modulated insulin action includes uptake of glucose and insulin antilipolytic action. AMP-activated protein kinase (AMPK) was vital in glucose and lipid metabolism, to increase CPT-1a mRNA expression. 6, 12 or 24 h of 40 µM sinensetin exposure markedly induced the phosphorylation of AMPK and acetyl-CoA carboxylase (ACC), increased phosphorylation of LKB1 and the level of CPT-1a mRNA. These results suggested sinensetin took part in the AMPK signaling pathway (Kang et al., 2013).

An imbalance between adipogenesis and lipolysis leads to obesity. Results reported by Kang et al. (2015) revealed enhancement of adipogenesis in preadipocytes and lipolysis in mature adipocytes. Treatment with 2, 10 and 40 µM sinensetin resulted in increment of cellular lipid accumulation and triglyceride content in a dose-dependent manner in 3T3-L1 cells. Somehow the effect was less than positive control which was treated with 0.5 mM 3-isobutyl-1-methylxanthine (IBMX). Sinensetin also upregulated expression of peroxisome proliferator-activated receptor (PPAR)γ1, PPARγ2, CCAAT/enhancer-binding protein (C/EBP) α and fatty acid-binding protein aP2. These results suggested sinensetin enhanced lipogenesis in preadipocytes 3T3-L1. Adiponectin, as a direct target of PPARγ increased significantly (*p* < 0.05) in a dose-dependent manner. Sinensetin possessed ability the same as IBMX to increase expression of differentiation signals, SREBP1c and C/EBPβ relative to DMSO-treated cell in 3T3-L1 preadipocytes. It also increased phosphorylation of cAMP-responsive element-binding protein, CREB and extracellular signal-regulated kinase ERK, significantly (*p* < 0.05) increase phosphorylated forms of PKA at 0.25 and 0.5 h compared with DMSO-treated cells. cAMP levels increased dose-dependently with 30 min treatment of sinensetin. These results suggested sinensetin enhanced lipogenesis in preadipocytes 3T3-L1 *via* cAMP/PKA pathway. In search of the effect of sinensetin on lipolysis, glycerol levels in culture supernatant were measured. It was reported that sinensetin significantly increased lipolysis at 24 and 48 h after treatment measured with glycerol level associated with an increment of cAMP at 0–40 µM in mature 3T3-L1 adipocytes (Kang et al., 2015).

### Antidiabetic Effect

Mohamed et al. (2013) demonstrated that fraction consisted mainly of sinensetin and eupatorin possessed the highest glucose-lowering effect in *O. aristatus.* It reduced glucose absorption of jejunum without changing the insulin level in streptozocin-induced rat. Hydrolysis of starch by pancreatic α-amylase and uptake of glucose by intestinal α-glucosidase caused hyperglycaemia in type 2 diabetes mellitus when there was a sudden increase in blood glucose level. Potentiation of α-glucosidase and α-amylase inhibition may be used as an effective strategy for post-prandial hyperglycemia linked to type2 diabetes mellitus. Mohamed et al. (2012) reported strong inhibition percentages (89.0–32.0%) for α-glucosidase at concentration range of 2.5–0.31 mg/ml. The activity was comparably strong with IC_50_ value of 0.66 mg/ml relative to acarbose (IC_50_ = 1.93 mg/ml, *p* < 0.01) and 50% ethanolic extract of *O. aristatus* (IC_50_ = 4.63 mg/ml, *p* < 0.01). However, Damsud et al. (2014) had different findings which showed no inhibition towards yeast α-glucosidase and rat intestinal α-glucosidases (maltase and sucrase) when 10 µL sinensetin was mixed with 0.1 U/mL α-glucosidase. In addition, sinensetin showed maximum inhibition of 85.8% at 2.5 mg/ml against porcine α-amylase (Mohamed et al., 2012). IC_50_ value of sinesetin (1.13 mg/ml) was significantly lower than both acarbose (IC_50_ = 4.89 mg/ml, *p* < 0.05) and 50% ethanolic extract of *O. aristatus* (IC_50_ = 36.70 mg/ml, *p* < 0.001). The provided *in vitro* evidence demonstrated appreciable inhibitory effect of sinensetin on α-amylase and α-glucosidase and generated a stronger rationale for further studies on sinensetin.

### Diuretic Effect

Since *O. aristatus* is used for several kidney disorders, gout and as diuretics traditionally, it was examined for its diuretic and hypoglycemic effects. One of the reports on the diuretic activity of *O. aristatus* described significant enhancement of ion excretion, especially potassium ion urinary excretion, although activity was lower than that of furosemide treated in Sprague–Dawley rats model (Arafat et al., 2008; Adam et al., 2009). Diuretic action is vital in kidney stone treatment, dissolving the stone when fluid volume flowing through kidney increases. Adenosine A_1_ receptor antagonist function as diuresis and sodium excretion inducer affords renal protection (Modlinger and Welch, 2003; Welch, 2015; Tofovic et al., 2016). Adenosine A1-receptor binding assay was also carried out on seven active methoxy flavonoids from *O. aristatus* (Yuliana et al., 2009). Sinensetin exhibited pK_i_ value of 5.5 µM which was comparable to thise of luteolin (pK_i_ = 5.4) and quercetin (pK_i_ = 5.8). All flavonoids including sinensetin, luteolin and quercetin demonstrated a one-site curve with a Hill slope value within 0.9–1.4, not significantly different with unity, indicating the antagonist effect.

### Vasorelaxant Effect

Vasorelaxant effect of sinensetin was reported by Yam et al. (2018) recently, employing *in vitro* pre-contraction aortic ring assay. It was suggested that the mechanism of action might be via NO/sGC/cGMP and indomethacin pathways, potassium and calcium channels, and muscarinic and beta-adrenergic receptors. Sinensetin at a range of 0.03–2.11 µM caused concentration-dependent vasorelaxation of 1 µM phenylephrine (PE) pre-contracted endothelium–intact aortic rings (pD2 = 6.97, *E*_max_ = 108%). There were still sinensetin-induced vasorelaxation in various conditions or pretreated with different antagonist and the results obtained from endothelium-denude (pD2 < 5.67, *E*_max_ = 31.38%), *L*-NAME (pD2 = 6.42, *E*_max_ = 75.46%), ODQ (pD2 = 6.18, *E*_max_ = 76.72%) and methylene blue (pD2 = 5.52, *E*_max_ = 52.69%) were significantly reduced (*p* < 0.001). Potassium channels involvement determined with the application of potassium blocker on endothelium-intact aortic 20 min before pre-contraction with PE. Propanolol as a β-blocker significantly (*p* < 0.05) decreased the vasorelaxation effect of sinensetin (pD2 = 6.71, *E*_max_ = 95.4%). Besides, sinensetin (0.04–0.58 µM) and calcium channel blocker, nifedipine (0.1–1.0 µM), significantly reduced the increase in contraction caused by the addition of 3 mM calcium ions, diminishing the effect of calcium ions induced contraction (*p* < 0.05) by 0.65–0.31 g and 0.1–0.03 g, respectively. Sinensetin depicted concentration-dependent vasorelaxant effect on endothelium-intact aortic rings. The evidence supported the suggestion that vasorelaxant effect of sinensetin did not solely dependent on endothelium but endothelium-independent relaxing factors might partially contribute.

### Antidementia

Nagase et al. (2005) isolated sinensetin from the peel of *C. reticulata* and reported on its antidementia activity using transient transfection and reporter gene assay. cAMP/protein kinase A (PKA)/Extracellular related kinase (ERK)/cAMP response element-binding protein (CREB) linked to CRE-mediated transcription is vital in the aspect of learning and memory (Kawahata et al., 2013). cAMP response element (CRE) transcription is dysregulated in neurodegenerative disorders in the central nervous system. Protective action against neurodegeneration in CNS includes stimulating CRE-dependent transcription and induce neurite outgrowth. Sinensetin from *C. reticulata* peel enhanced CRE-dependent transcription (*p* < 0.01) after 5 h treatment at 100 µM in PC12D cells. In conjunction, 100 µM sinensetin exposition for 48 h induced neurite outgrowth (*p* < 0.01). Among the compounds tested nobiletin and 6-demethylnobiletin were more potent than sinensetin (Nagase et al., 2005). However, Kawahata et al. (2013) found that sinensetin isolated from *C. reticulata* peel enhanced transcription to a greater extent than nobiletin at concentration up to 30 µM (*p* < 0.001) tested on CRE-mediated transcription in cultured hippocampal neuron from embryos of an 18-days pregnant Sprague-Dawley rat. Sinensetin exhibited the most potent activity among the tested compounds (tangeretin, 6-demethoxynobiletin, 6-demethoxytangeretin and nobiletin) with *p* value <0.01 as compared to nobiletin, facilitating the transcription. The discrepancy between the results of both studies might be due to the cell-type-dependent difference in the response of these compounds. Meanwhile, Kimura et al. (2013) studied the effect on ERK phosphorylation in PC12D cells. No significant result was observed when cells were stimulated with sinensetin. Previous studies have suggested the generation of intraneuronal amyloid-beta (Aβ) oligomers is an early event in the pathogenesis of Alzheimer’s disease (Kimura et al., 2013). Beta-site amyloid precursor protein (APP) cleaving enzyme1 (BACE1) catalyzes the rate-limiting step of Aβ generation, considered as a prime target for Alzheimer’s disease (Youn et al., 2017). Sinensetin inhibited BACE1 with an IC_50_ value of 6.3 × 10^–5^ M, to lower Aβ generation in a dose-dependent manner (*p* < 0.001) with K_i_ value of 3.8 × 10^–5^ M. Tangeretin (4.9 × 10^–5^ M) has the highest potency followed by nobiletin (5.9 × 10^–5^ M) and sinensetin. Inhibition of sinensetin was specific, exhibiting no statistically significant inhibition against TACE and other serine proteases (trypsin, chymotrypsin, elastase) (Youn et al., 2017).

### Antimicrobial Effect

When 19 citrus cultivar were investigated for their antitrypanosomal activities to tackle African trypanosomiasis such as *Trypanosoma brucei, T. brucei gambiense* and *T. brucei rhodesiense*, “Setoka” extract exhibited the most potent inhibitory effect (36%) on *T. brucei* proliferation with highest activity in its ethyl acetate extract (Nakanishi et al., 2019). “Setoka” is one of the second-generation hybrids between “Kiyomi” (*C. reticulata*) and other *Citrus* species. All of the compounds subjected to the parasite inhibition assay showed potent activities against *T. brucei* in a dose-dependent manner. Pentamidine (IC_50_ = 0.001 μg/ml) used as a standard was the most potent, followed by nobiletin (IC_50_ = 2.4 μg/ml), reagent grade nobiletin (IC_50_ = 3.1 μg/ml), reagent grade sinensetin (IC_50_ = 4.1 μg/ml) and sinensetin (IC_50_ = 4.8 μg/ml). 293T human embryonic kidney cell was employed to assess toxicity against mammalian cells by resazurin assay. Selectivity was calculated for all these compounds based on IC_50_ values against *T. brucei* and 293T human embryonic kidney cells. Descending sequence of selectivity was as follows; pentamidine (SI = 3,400), nobiletin (SI = 5.4), reagent grade nobiletin (SI = 4.5), reagent grade sinensetin (SI = 2.9), sinensetin (SI = 2.7), HMF (SI = 1.3). Taken together all the data reported, sinensetin and nobiletin were responsible for antitrypanosomal activity in “Setoka” peel. Unfortunately, the IC_50_ values and selective indexes were not comparable with those of modern antitrypanosomal candidates. Vikram et al. (2010) evaluated quorum sensing, biofilm and type three secretion system (TTSS) inhibitory properties of citrus flavonoids including sinensetin using *in vitro* model. Sinensetin dose-dependently inhibited AI-2- mediated bioluminescence, demonstrating maximal response at 100 μg/ml in *Vibrio harveyi* mutant strain MM32 (AI-2). Meanwhile, sinensetin strongly inhibited biofilm formation in *E. coli* O157: H7 and *V. harveyi* without inhibiting the growth of *V. harveyi* and *E. coli* O157: H7. The results were proposing inhibition of A1-mediated cell-cell signalling by sinensetin lead to antagonistic activity against biofilm formation. Continuation of work further discovered 100 µM of sinensetin significantly (*p* < 0.05) inhibited motility halos of *E. coli* O157: H7 by measuring zone diameter after culture of EHEC was stabbed in 0.3% LB-agar plate for 12 and 24 h (Vikram et al., 2013).

## Toxicology

The medicinal benefits of sinensetin have raised the interest of many researchers to further study its pharmacological and toxicological properties. The results of toxicological studies are listed in Table 2. For instance, Lam et al. (2012) reported the lowest lethal toxicity of sinensetin as compared to nobiletin and hesperetin by using zebrafish embryo. Beating heart rate of zebrafish embryo was the parameter used to indicate the living status of embryo. The toxicity of the compounds at concentration of 30, 100 and 300 µM for 48 h was determined. At 48 h post-treatment with 100 µM sinensetin, about 40% of embryos were reported alive, achieving a 94.6% survival rate. However, Nakanishi et al. (2019) revealed non-negligible cytotoxic effect of sinensetin and reagent grade sinensetin against 293T human embryonic kidney cells with IC_50_ values of 11.2 and 13.8 μg/ml, respectively, by a resazurin assay *in vitro*. Most of the cytotoxic effects conducted reported minimal cytotoxicity, as compared to other tested compounds and untreated control. Sinensetin at 20 µM demonstrated 84.5% growth of K562/ADM cells when 10 µL WST-1 was used to evaluate cell viability treated for 48 h (Ohtani et al., 2007). The result was a ratio relative to that in the absence of vincristine, indicating not much different from the untreated control (85.3%). Sinensetin exerted toxicity against AML-2/D100 cells with an IC_50_ value of more than 400 µM while the effective dose for its synergistic chemosensitizing effect with vincristine has an IC_50_ value of 3.2 µM (Choi et al., 2004). No activity was observed too as in the study conducted by Jeong at al. (2007) up to concentration of 400 µM. These studies indicate that sinensetin exerted minimal toxicity to normal cells and possessed high selectivity.

**TABLE 2:** Toxicity of sinensetin.

Plant name/Source	Plant part	Assay type	Activity	Conclusion	References
*Citrus reticulata* Blanco	Peel	*In vitro*	Cytotoxicity	Exert relatively low cytotoxicity in AML-2/100 (IC_50_ = 82.4 µM) with lower hemolytic effect on RBC than other flavonoids	Choi et al. (2002)
–	–	*In vitro*	Cytotoxicity	Exert minimal toxicity (IC_50_ > 400 µM) as compared to other tested flavonoids in AML-2/D 100 cells	Choi et al. (2004)
Cultivar Setoka Synthetic: Funakoshi	Peel ethanolic extract	*In vitro*	Cytotoxicity	Possess toxicity against 293T human embryonic kidney cells with IC_50_ = 13.8 ± 3.8 μg/ml, IC_50_ = 11.2 ± 0.4 μg/ml with selective index (SI) of 2.9 and 2.7, respectively, which is non-negligible	Nakanishi et al. (2019)
Synthetic: ChromaDex	–	*In vitro*	Cytotoxicity	No toxicity up to 20 μM in human mast cell (HMC-1) cell	Chae et al. (2017)
Synthetic: Extrasynthese	–	*In vitro*	Cytotoxicity	10 µL WST-1 solution is used to evaluate growth inhibition on cells treated with 20 µM sinensetin and 1 µM vincristine for 48 h, resulted in 84.5 ± 6.4% growth of K562/ADM cells relative to that in the absence of vincristine, the result is not much different with the untreated control (85.3 ± 3.5%)	Ohtani et al. (2007)
Synthetic: J & K Scientific Co.	–	*In vitro*	Cytotoxicity	100 µM sinensetin is nontoxic after 4 h exposure on MDR1-MDCK II cells measured with MTT assay	Bai et al. (2019)
Synthetic: Indofine	–	*In vitro*	Cytotoxicity	No activity up to 400 µM towards AML-2/D100 cells	Jeong et al. (2007)
Synthetic: Shanghai Tauto Biotech Co.	-	*In vitro*	Cytotoxicity	Lowest lethal toxicity among test compunds in embryos with about 40% of embryos were alive at 48 hpt with 100 μM sinensetin, achieved 94.6 ± 2.1% survival rate	Lam et al. (2012)

## Conclusion

Undeniably, in recent years, there is an elevating global demand in natural products for healthcare and also finding leading compounds for several diseases in drug discovery and development. The growing demand and significance of natural products and compounds should not be underestimated. Sinensetin was studied and found to be possessing a diversity of beneficial pharmacological effects such as anticancer, antidiabetic, antidementia, vasorelaxant, antimicrobial and anti-inflammatory activities. Since 1989, people had paid much attention to its anticancer activity. Many *in vivo* and *in vitro* studies had indicated that sinensetin not only showed good activity towards tumor cells, but also exert minimal toxicity to normal cells according to the ratio of IC_50_ values, and possessed high selectivity. Thus, we conclude sinensetin is a promising anticancer drug candidate for future development to delay cancer progression and prolong survival. Since we are still lacking effective anticancer drug with good safety profile, potential of sinensetin will be worth studying. However, most of the mechanisms are poorly understood for all activities aforementioned. Therefore, more investigation and further studies of mechanisms of action need to be performed before it can be used as a therapeutic drug in the future. There is a necessity for performing toxicology profile of sinensetin *in vivo* in future research to further evaluate its safety profile because as shown in Table 2, all the toxicity studies were performed *in vitro*. The ability of sinensetin to aid in delaying cancer progression is definitely worth further studying with the evidence of IC_50_ values and selectivity index in previous studies as aforementioned. Focusing on the anticancer ability of sinensetin, more supporting studies should be carried out such as the exact mechanisms and all the genes and extend of genes expression to further develop sinensetin as a lead compound.
